# Inflammatory markers and body mass index amoung hispanic children

**DOI:** 10.1371/journal.pone.0289523

**Published:** 2024-06-28

**Authors:** Henry Lang, Elaine N. Loudermilk, W. Andrew Clark, Jo-Ann Marrs, T. Andrew Joyner, Liang Wang, Kathryn S. Gerber, Arsham Alamian

**Affiliations:** 1 College of Medicine, Oklahoma State University, Stillwater, OK, United States of America; 2 Public Health, 30^th^ Operational Medical Readiness Squadron, 30^th^ Medical Group, Vandenberg Space Force Base, CA, United States of America; 3 College of Clinical and Rehabilitative Health Sciences, East Tennessee State University, Johnson City, TN, United States of America; 4 College of Nursing, East Tennessee State University, Johnson City, TN, United States of America; 5 Department of Geosciences, College of Arts & Sciences, East Tennessee State University, Johnson City, TN, United States of America; 6 Robbins College of Health and Human Sciences, Baylor University, Waco, TX, United States of America; 7 School of Nursing and Health Studies, University of Miami, Coral Gables, FL, United States of America; Università degli Studi di Milano, ITALY

## Abstract

**Background and objectives:**

Body mass index (BMI) is inversely proportional with adiponectin levels among adults, while insulin, C-reactive protein (CRP), interleukin 6 (IL-6), resistin and tumor necrosis factor-alpha (TNF-α) have been linked with elevated BMI. The role and relation of these biomarkers with BMI among a Hispanic pediatric population are less known. Thus, the objective of this study was to examine the association of inflammatory markers with the odds of overweight/obesity while controlling for several sociodemographic factors among a Hispanic youth population in Northeast Tennessee.

**Methods:**

Height, weight, demographic information, and blood samples were collected from 107 Hispanic children aged 2 to 10 years recruited at a large community health center in 2015–2016 in Northeast Tennessee. Data for this research were accessed and analyzed in 2022. Multivariable logistic regression was conducted to assess the relations between adiponectin, insulin, resistin, CRP, TNF-α, and IL-6, and overweight/obesity vs. having a healthy (normal) weight.

**Results:**

Adiponectin levels were significantly lower among overweight/obese Hispanic children (p = 0.0144) compared to healthy weight children. The odds of overweight/obesity decreased by 4% for every one-unit increase in serum adiponectin. Insulin levels were significantly higher among overweight/obese Hispanic children compared to healthy weight children (*p =* 0.0048). The odds of overweight/obesity increased by 7% for every one-unit increase in serum insulin. Resistin, IL-6, TNF-α, and CRP were not significantly associated with overweight/obesity in this population.

**Conclusion:**

Adiponectin behaves similarly in Hispanic youth as it does in other pediatric populations, possibly making it a valuable marker when examining metabolic health status in this population.

## Introduction

The prevalence of obesity among youth ages 2 to 19 years old in the United States (U.S.) increased from 17.7% in 2011–2012 to 21.5% in 2017–2020 [1]. Increasing prevalence of obesity among children is concerning due to the shared relationship of childhood and adult obesity; children with obesity are at a greater risk to have obesity during adulthood than children with normal weight [2, 3]. Childhood obesity in turn increases risk for metabolic conditions such as type II diabetes mellitus (T2DM), atherosclerosis, stroke, sleep apnea, respiratory disorders, and certain cancers [2–4]. Childhood obesity is a multifaceted disease, with many contributing factors including social economic status (SES) and metabolic alterations. Hispanic populations in the United States have been identified as having a greater risk for obesity compared to the general population, due to a combination of SES and metabolic factors [5–7]. Due to complexity of childhood obesity in underrepresented populations, the aim of this study was to include variables encompassing both the SES and biochemical aspects of the disease.

Maternal education level is a specific SES variable thought to have an inverse relationship with childhood obesity [8]. Literature has demonstrated disparities between maternal education in the Hispanic community when compared to the general population, which may contribute to the increased incidence of obesity in the Hispanic population [5, 9]. However, SES variables alone are not sufficient in predicting obesity risk and complications at the individual patient level [10]. Determining risk in individuals with obesity by analyzing biochemical markers for the development of childhood T2DM and additional associated conditions allows for early prevention interventions, which may improve metabolic status throughout life [11, 12].

Insulin, an inhibitory regulator of gluconeogenesis, is commonly monitored in the clinical setting to determine metabolic response to dietary glucose intake [13]. Chronically elevated insulin levels are associated with a reduction in adiponectin [14]. However, diet may cause fluctuations in insulin requiring pre-measurement fasting to acquire accurate values [13]. Adiponectin, another inhibitor of gluconeogenesis, functions upstream of insulin and may be a valuable marker in determining insulin sensitivity, while possibly demonstrating less postprandial fluctuations than insulin [15, 16]. Adiponectin has also been demonstrated to be an independent and earlier predictor for metabolic health status when compared to insulin [17].

Adiponectin has been demonstrated to predict obesity and metabolic health status among adult populations, and is a potential target for personalized medical therapy, as adiponectin may contribute to obesity and diabetes to varying degrees between and within populations [18]. The Hispanic population represents a historically underrepresented group in academic medicine, making them an important target of additional research in the potential rise in personalized medicine [19].

Previous studies have shown body mass index (BMI) to be inversely proportional with adiponectin levels among adults and children [20, 21]. Increased levels of adiponectin have been associated with an increase in insulin sensitivity, increasing glucose uptake in peripheral tissue and leading to reductions in glycogenolysis and hepatic gluconeogenesis [22]. The downregulation of hepatic gluconeogenesis is coupled with an increase in fatty acid β-oxidation, leading to improved lipid profiles [23]. Treatment with adiponectin in rats demonstrated an increase in insulin-stimulated glucose uptake in adipose tissue through the upregulation of adenosine monophosphate protein kinase (AMPK), reducing the phosphorylation of p70 S6 kinase, which acts as an inhibitor [24].

Conversely, C-reactive protein (CRP), interleukin 6 (IL-6), resistin, and tumor necrosis factor-alpha (TNF-α) are associated with elevated BMI, inflammation, and poor metabolic status; these markers have also been linked to increased risk for developing T2DM and atherosclerotic lesions, and have been found to be inversely proportional to adiponectin [25–27]. The relationships between adiponectin, BMI, and insulin have been explored among children [28, 29]. However, there remains a gap in the literature regarding the relationship of adiponectin, insulin, CRP, IL-6, resistin, and TNF-α with BMI among Hispanic children. Thus, the objective of this study was to examine the association between inflammatory markers and the likelihood of being overweight/obese while controlling for sociodemographic factors within Hispanic youth in Northeast Tennessee.

## Methods

### Participants

Secondary analyses of height, weight, demographic information, and blood draws from 114 Hispanic children aged two to ten years recruited in 2015–2016 at a large community health center in Northeast Tennessee (as part of a larger study on metabolic syndrome) [30] were completed. Data for this article were accessed for research purposes and analyzed from September to December 2022. Of the 114 children considered, 1 was excluded due to having a BMI below the 5^th^ percentile (i.e., underweight), 2 had missing data on maternal education, and 4 had missing information on TNF-α, resistin and adiponectin. The final analytic sample size comprised of 107 children. Methods for the collection of data used are mentioned elsewhere in a study conducted by Alamian et al. [30] The authors did not have access to information that could identify individual participants during or after data collection.

This study received approval from the East Tennessee State University Human Subject Research Ethics Committee (IRB#: 0414.16s).

### Dependent variable

The outcome of interest, BMI status (obese defined as overweight/obese vs. healthy weight, according to 2000 CDC growth charts) [31], was treated as a categorical variable.

### Independent variables

The variables of interest were run in duplicate, and included adiponectin (*μ*g/mL), insulin (uIU.L), resistin (pg/mL), TNF-*α*(pg/mL), CRP (mg/dL), and IL-6 (pg/mL). Analysis for adiponectin (171A7002M), insulin and resistin (171A7001M), and TNF-*α* and IL-6 (171A7002M) were performed using Bio-Rad Bio-Plex Mag-Pix according to provider procedures. East Tennessee State University Clinical Laboratory: an accredited reference lab (Center for Medicare & Medicaid Services Clinical Laboratory, certification number 44D0659180) was utilized for analysis of serum CRP.

### Covariates

Covariates included child age, child sex (male or female), maternal education level (high school graduation or more vs. less than high school education), maternal marital status (married versus other). Child age was reported as a continuous variable in years.

### Statistical analysis

Descriptive statistics (frequencies and percentages for categorical variables, and means and standard deviations for continuous variables) were performed to describe the data as appropriate. Pearson chi-squared test and independent *t*-tests were conducted to examine differences in percentages (for categorical variables) and means (for continuous variables) by weight status (overweight/obese versus healthy weight status). Post-hoc analyses were conducted to verify the influence of outliers on descriptive and inferential statistics. No major influence on results was noted after potential extreme values were excluded. As a result, and following Central Limit Theorem, analyses were conducted on the full sample after removing missing variables. Benjamini-Hochberg adjustments were completed to control for inflated type I error due to multiple comparisons [32]. Simple and multiple logistic regression analyses were then performed to assess the strength of association between variables of interest and being overweight/obese versus healthy weight among participants in this population. Multiple logistic regression models controlled for the potential effects of covariates (age, sex, maternal education, maternal marital status). Statistical analysis was completed via statistical analyst system (SAS, version 9.4; SAS Institute, Cary, NC).

## Results

The majority of the study sample were female (54.21%) with an average age of 6.62 years (SD: 2.74 years) as seen in Tables 1 and 2. Almost half of the sample were classified as overweight/obese (44.86%) with the remaining sample having a healthy weight status (55.14%). Slightly over half of mothers had less than a high school education (56.07%) and most were married (78.50%). A greater percentage of males (48.98%) were overweight/obese compared to females (41.38%), although this difference was not statistically significant. There were no statistically significant differences in weight status by maternal education or marital status (P > 0.05). Average levels of biomarkers were as follows: Adiponectin, 24.27 *μ*g/mL (SD = 13.85); insulin, 16.03 uIU.L (SD = 17.46); resistin, 5510.23 pg/mL (SD = 3817.13); TNF-*α*, 8.40 pg/mL (SD = 17.00); CRP, 2.35 mg/dL (SD: 4.86); and IL-6, 3.23 pg/mL (SD = 8.68).

**Table 1:** Sociodemographic characteristics of the Hispanic pediatric sample by healthy and overweight/obese weight status (n = 107)^a^.

	N (%) Total	Healthy Weight	Overweight/Obese	P-value^b^
**n, %, Total**	107 (100.00)	59 (55.14)	48 (44.86)	
**Sex, n (%)**				0.4310
Male	49 (45.79)	25 (51.02)	24 (48.98)	
Female	58 (54.21)	34 (58.62)	24 (41.38)	
**Maternal education, n (%)**				0.6711
Less than high school education	60 (56.07)	32 (53.33)	28 (46.67)	
High school graduate or more	47 (43.93)	27 (57.45)	20 (42.55)	
**Maternal marital status, n (%)**				0.2044
Married	84 (78.50)	49 (58.33)	35 (41.67)	
Other	23 (21.50)	10 (43.48)	13 (56.52)	

a. Data from a study of metabolic syndrome in Hispanic children in Johnson City, TN [19].

b. P-value from the chi-squared test.

**Table 2:** Mean (SD) for child age and selected biomarkers by healthy weight and overweight/obese weight status among the Hispanic pediatric sample (N = 107)^a^.

	Healthy Weight (N = 59)		Overweight/Obese (N = 48)		
	Mean	Std. Dev.	Mean	Std. Dev.	P-value^b^
Child age (years)	6.39	2.75	6.90	2.73	0.3446
Adiponectin (ug/mL)	27.53	15.37	20.27	10.56	0.0144
Insulin (uIU.L)	10.61	9.94	22.70	22.00	0.0048
Resistin (pg/mL)	4916.50	2387.40	6240.00	4984.10	0.1922
TNF-alpha (pg/mL)	8.18	13.23	8.67	20.88	0.9626
C-reactive protein (mg/dL)	2.33	5.48	2.38	4.01	0.9626
Interleukin 6 (pg/mL)	3.73	10.47	2.62	5.84	0.7332

a. Data from a study of metabolic syndrome in Hispanic children in Johnson City, TN [19].

b. P-value from t-test adjusted by Benjamini-Hochberg method in order to correct for multiplicity of hypotheses.

Results from the independent *t*-tests for differences in biomarkers by weight status are shown in Table 2. Adiponectin levels were significantly lower among overweight/obese children compared to healthy children (*P* = 0.0144). Insulin levels were significantly higher among overweight/obese children compared to healthy children (*P =* 0.0048). No other significant differences were observed between overweight/obese and healthy children when considering other biomarkers or the child’s age.

Simple logistic regression (SLR) and multiple logistic regression (MLR) results are presented in Table 3. In SLR models, both adiponectin and insulin were significantly associated with being overweight/obese. For every one-unit increase in serum adiponectin, the child’s odds of overweight/obesity decreased by 4% [Odds Ratio (OR): 0.96, 95% confidence interval (95% CI): 0.93–0.99]. In contrast, every one-unit increase in insulin resulted in a 7% increased chance of overweight/obesity (OR: 1.07, 95% CI: 1.03–1.11). MLR results corroborated the SLR findings: both adiponectin and insulin were found to be significantly associated with overweight/obesity. For every one-unit increase in serum adiponectin, the odds of overweight/obesity decreased by 4% (OR: 0.96, 95% CI: 0.93–0.99) while holding all other variables constant. For every one-unit increase in insulin, the odds of overweight/obesity increased by 7% (OR: 1.07, 95% CI: 1.02–1.13). Child age, child sex, maternal education and marital status, and other biomarkers (resistin, IL-6, TNF-α, and CRP) were not significantly associated with overweight/obesity.

**Table 3:** Simple and multiple logistic regression examining the relationship between biomarkers and overweight/obesity in a Hispanic pediatric sample from Northeast TN (N = 107).

	SLR^a^			MLR^a^		
Variables	OR^b^	95% CI^c^	P-value	OR^b^	95% CI^c^	P-value
Adiponectin (ug/mL)	0.96	0.93–0.99	0.0084	0.96	0.93–0.99	0.0104
Insulin (uIU.L)	1.07	1.03–1.11	0.0017	1.07	1.02–1.13	0.0052
Resistin (ug/mL)	1.00	1.00–1.00	0.1000	1.00	1.00–1.00	0.0477
TNF-alpha (pg/mL)	1.00	0.98–1.02	0.8813	1.01	0.95–1.08	0.6748
C-reactive protein (mg/dL)	1.00	0.93–1.08	0.9632	0.99	0.90–1.09	0.8879
Interleukin 6 (pg/mL)	0.98	0.93–1.04	0.5235	0.91	0.75–1.09	0.2867
**Child sex**						
Female vs male	0.735	0.34–1.59	0.4314	0.71	0.28–1.82	0.3041
**Child age** (years, continuous)	1.28	0.58–2.83	0.3415	0.82	0.31–2.13	0.8268
**Maternal education level**						
High school education or less	Reference			Reference		
High school graduate or more	0.85	0.39–1.83	0.6712	0.67	0.27–1.70	0.4310
**Maternal marital status**						
Other vs married	1.82	0.72–4.62	0.2077	1.58	0.53–4.70	0.4592

Simple logistic regression (SLR), multiple logistic regression (MLR)

Odds Ratio

95% Confidence Interval

## Discussion

Results of this study indicate that both adiponectin and insulin levels are potentially significant predictors of childhood overweight/obesity. Adiponectin remains protective against increased BMI when accounting for insulin. Results from this study confirm findings from similar studies that demonstrated significantly lower adiponectin levels in Italian and Pima Native American children with overweight/obese classification using BMI [28, 29]. The repeated findings of low adiponectin in overweight/obese children may be related to the relationship between adiponectin and insulin. Depressed adiponectin concentration and chronically elevated insulin have been demonstrated in the pathogenesis of insulin resistance, and therefore metabolic syndrome [33, 34]. The depressed levels of adiponectin found in overweight/obese children, coupled with participant age and the lack of significant differences between groups in the other biomarkers examined, suggests adiponectin levels may be an early indicator to predict risk of developing insulin resistance and metabolic syndrome in children, compared with other alternatives in the literature. While BMI has also been used as an early predictor of metabolic syndrome in children [35], research output is increasing regarding metabolically healthy (MHO) vs metabolically unhealthy (MUO) individuals with obesity, shifting the focus of intervention trials towards improved metabolic health status as the desired outcome rather than weight loss alone. Alterations in BMI, while helpful, are not required following life-style intervention for metabolic health to improve [36, 37]. Adiponectin may be a valuable variable to monitor when tracking the effects an individual’s BMI may have on their metabolic health (i.e., if an individual has high adiponectin and elevated BMI, there may be less concern for weight-loss intervention) [38]. Longitudinal studies controlling for BMI while monitoring insulin sensitivity and adiponectin in children are needed to confirm this hypothesis.

The lack of significance of resistin, CRP, IL-6, and TNF-α between the normal BMI and overweight/obese groups may be due, in part, to the pathological progression of obesity. The infiltration of inflammatory cells into adipocytes is thought to occur after a decrease in adiponectin [39], suggesting the duration of overweight/obesity is a key factor in the progression of chronic inflammation. The participants in the current study may not have had overweight/obesity for a sufficient length of time to elicit the inflammatory consequences typically associated with obesity in adulthood [40]. Previous research regarding resistin levels in the adult population is mixed, with some findings demonstrating no differences in individuals with obesity compared to those of healthy weight, whereas other data demonstrated significantly greater resistin levels in populations with obesity [41, 42]. Additional literature regarding the ability of the inflammatory markers CRP, IL-6, and TNF-α to predict overweight/obesity status has also led to mixed results, leading some researchers to suggest the relationship may be age dependent [43, 44]. The findings from the current study indicate adiponectin may be an earlier predictor for the risk of developing insulin resistance compared to CRP, IL-6, and TNF-α in the pediatric population. Further research in the Hispanic pediatric population is required to confirm the findings of the current study.

The adjusted analysis in this study suggests a negative association between maternal education and BMI, however the findings were not significant. Previous research has demonstrated parental education to be a predictor of child overweight/obese status in countries with a high economic status. Lê-Scherban and colleagues (2021) revealed childhood obesity prevalence to be greater among children whose parents did not obtain a high school education compared to children with parents who had a college degree [45]. Ogden and colleagues (2018) found childhood obesity prevalence to be significantly lower in Hispanic US households when the head of the household was a college graduate compared to a high school graduate or less [46].The current study examined the effects of maternal completion of high school on childhood obesity, which may elicit a smaller effect than when comparing maternal non-high school graduates to college graduates.

The current study was limited by a small sample size, possibly leading to a lack of statistical power. Future studies including greater statistical power could improve the accuracy of the relationship between variables. However, significance was still found despite the limited sample size, indicating the relationships uncovered should be studied further. Furthermore, the cross-sectional design of the study precluded making causal claims. The study’s sample population was of a specific region in Tennessee where obesity is known to be prevalent [47], thus additional studies should seek to examine similar populations in urban, suburban, and rural settings to improve external validity.

## Conclusion

In summary, this study revealed adiponectin behaves similarly in Hispanic children as it does among adults potentially making it a valuable marker when examining health status among Hispanic pediatric populations. Resistin, CRP, IL-6, and TNF-α may not be relevant markers to predict overweight/obese status in Hispanic youth, as these markers may not become consistently elevated until an individual has maintained overweight/obese status for an extended length of time. Longitudinal studies are needed to better understand the relationship, and determine the clinical significance, between Hispanic youth BMI and adiponectin, resistin, CRP, IL-6, and TNF-α. The degree to which maternal education influences childhood obesity in the Hispanic populations should be further explored, using larger sample sizes while including biochemical markers shown to predict obesity and additional metabolic complications.
